# 

**Published:** 1847-06

**Authors:** 


### CONTENTS.

### LIBRARY.

Complete Elements of the Science and Art of the Dentist. Translated from the French of M. Desirabode, by *—A—*,........ .... 305

### JOURNAL.

ART. I.—Address to the Graduating Class of the Baltimore College of Dental Surgery, by Eleazar Parmly, M. D.,... ........... 297
ART. II.—Observations upon the Importance and Value of a Knowledge of the Collateral Branches of Medicine and Surgery in connection with Dentistry. By James Robinson, D. D. S., 324
ART. III.—The Teeth a Test of Age, considered with reference to the Factory Children. By Edwin Saunders, D. D.S., &c., &c., 330
ART. IV.—Letter from Dr. W. H. Dwinelle,..... .. ............. 375
ART. V.—Observations on Congenital Fissure of the Palate, with some Remarks on Articulation and Impediments of Speech. By Charles W. Stearns, Esq., Surgeon, London,............ 377

### COLLECTANEA.

On the Treatment of Neuralgia by Tobacco,...................... 323
On a Simple Means of Arresting Bleeding from Leech Bites,........ 329
Polypus of the Nose and Ear,.................................... 388
Caries of Left Temporal Bone,................................... 389
New Method of Treating a Professional Rival,........................ 389
Offer of a Bribe for a good Review by a Dentist,...... ......... ... 390
Digestion of Alcoholic Fluids,.................................. 391
Case of Atrophy following an Injury of the Right Superior Maxillary Nerve,................... ........................... 391
On the Injurious Effects of Camphor on the Teeth,................ 392
Case of Trismus,............................ ................ 393
Cancer of the Bones,.................................... ......... 395
Eighth Annual Meeting of the American Society of Dental Surgeons, 396
Ethereal Vapor,.......................................... .... 396

